# Development and confirmation of humanized plasma and epithelial lining fluid exposures of meropenem, cefiderocol and tobramycin in a standardized neutropenic murine pneumonia model

**DOI:** 10.1093/jac/dkae432

**Published:** 2024-12-05

**Authors:** Andrew J Fratoni, Alissa M Padgett, Hanna F Roenfanz, Erin M Duffy, David P Nicolau

**Affiliations:** Center for Anti-Infective Research and Development, Hartford Hospital, Hartford, CT, USA; Center for Anti-Infective Research and Development, Hartford Hospital, Hartford, CT, USA; Center for Anti-Infective Research and Development, Hartford Hospital, Hartford, CT, USA; CARB-X, Boston, MA, USA; Center for Anti-Infective Research and Development, Hartford Hospital, Hartford, CT, USA

## Abstract

**Background:**

Murine pneumonia models play a fundamental role in the preclinical development of novel compounds seeking an indication for the treatment of pneumonia. It is vital that plasma exposures in these models are not used as a surrogate for exposure in pulmonary epithelial lining fluid (ELF). Herein, human-simulated regimens (HSRs) in both plasma and ELF of meropenem, cefiderocol and tobramycin are described in the standardized COMBINE murine neutropenic pneumonia model.

**Materials and methods:**

HSRs were developed in both plasma and ELF for meropenem and cefiderocol as 2 g q8h 3 h infusions, and tobramycin 7 mg/kg 30 min infusion. Pharmacokinetic studies were performed to confirm plasma and ELF exposures in mice that recapitulated %*f*T > MIC for meropenem and cefiderocol, and *f*Cmax and *f*AUC_0-24_ for tobramycin in humans.

**Results:**

Concentration–time profiles and relevant pharmacodynamic exposures for all three compounds were well matched in mice relative to humans. None of the plasma HSRs were able to appropriately humanize the ELF. Thus, modifications of the plasma HSRs were necessary to provide unique HSRs specific to ELF exposure for each compound.

**Conclusions:**

It should not be assumed that lung penetration in mice relative to humans is equivalent. With HSRs confirmed for these three drugs with established clinical use in the treatment of patients with pneumonia, these humanized exposures within the standardized model will allow for back-translation of anticipated efficacy and provide purposeful quantitative benchmarks for cfu/lung assessments for researchers on an international scale.

## Background

Preclinical animal infection models continue to play an important role in the development of novel antibacterial compounds.^1^ However, many factors can either hinder or improve the clinical translation of the models and therefore the subsequent utility of the derived data. While the murine neutropenic thigh infection model remains the ‘gold standard’ for initial pharmacokinetic/pharmacodynamic (PK/PD) assessment, it is increasingly important for compounds seeking an indication for pneumonia to undergo assessment in the murine neutropenic pneumonia model.^2^ A compound that best exemplifies the relevance of these additional lung investigations is daptomycin, which is inactivated by alveolar surfactant. Unfortunately, this was not discovered until after clinical failure when used to treat severe community-acquired pneumonia during registrational trials.^3^

Akin to the need for accounting for interspecies differences in plasma protein binding in preclinical animal infection models, there is the need to consider interspecies differences in penetration at the site of infection. This is especially true for lung penetration into the epithelial lining fluid (ELF), as the rate and extent of system hysteresis can be significantly mismatched. Therefore, humanizing free plasma exposure in a murine pneumonia model cannot be assumed to achieve clinically translational exposures in the lung, which may be either relatively over- or underexposed.^2^

Further complicating the preclinical landscape of animal infection models is a lack of uniformity between laboratories and research groups.^4,5^ To alleviate this concern, the European Innovative Medicines Initiative-funded Collaboration for prevention and treatment of MDR bacterial infections (COMBINE) consortium has defined critical elements of the murine neutropenic pneumonia model in a proposed standardized protocol.^6^ The purpose of the studies described here was to use the COMBINE murine neutropenic pneumonia model to develop and confirm human-simulated dosing regimens (HSRs) for three commonly used antibiotics used to treat critically ill patients with pneumonia: meropenem, cefiderocol and tobramycin. HSRs were created to approximate not only the free plasma exposure, but also the lung ELF, with the hypothesis that different dosing regimens would probably be required to humanize each matrix based on interspecies differences in system hysteresis. Future investigations are planned to use these HSRs against a previously well-defined challenge set of Gram-negative isolates (*Klebsiella pneumoniae* and *Pseudomonas aeruginosa*) to define quantitative benchmarks for cfu/lung in the standardized model that could be replicated and utilized by research laboratories internationally.^7^

## Materials and methods

### Antimicrobial agents

Commercial vials were acquired as follows: meropenem 1 g (PremierProRx, Lot #0004E21), cefiderocol 1 g (Shionogi, Lot #0021) and tobramycin 80 mg/2 mL (Mylan, Lot #7608713). Vials were reconstituted as necessary per the manufacturers’ instruction and further diluted with normal saline to achieve concentrations required to deliver weight-based dosing to the mice.

### Bacterial isolates

*K. pneumoniae* CDC 851 was sourced from the CDC and FDA Antibiotic Resistance Isolate Bank (Atlanta, GA, USA) and stored locally frozen at −80°C in skim milk. Before pharmacokinetic studies, CDC 851 was sub-cultured twice on Trypticase soy agar with 5% sheep blood (Becton Dickinson and Co., Sparks, MD, USA) and incubated at 37°C for ∼16 h. Bacterial colonies from the overnight culture plate were suspended in NS to a McFarland target of ∼1.25 to produce the final inoculum. CDC 851 (meropenem MIC ≤0.12 mg/L, tobramycin MIC 16 mg/L, cefiderocol MIC undetermined) has the following known resistance mechanisms: EMRD, fosA5, KDEA, oqxA, oqxA, oqxB19, oqxB19 and SHV-11.^8^

### Ethics

Animals were maintained and utilized in accordance with National Research Council recommendations. The study protocol was reviewed and approved by the Institutional Animal Care and Use Committee at Hartford Hospital (Assurance #A3185-01).

### Laboratory animals and the neutropenic pneumonia model

The model followed the COMBINE protocol with laboratory-specific detailed methods as follows.^6^ Specific pathogen free CD-1, female mice 6–8 weeks old were acquired from Charles River Laboratories, Inc. (Raleigh, NC, USA). All animals were allowed to acclimatize for 72 h before any study procedures and were housed as groups of six at controlled room temperature in HEPA-filtered cages (Innovive, San Diego, CA, USA). Study rooms were maintained with diurnal cycles (12 h light/12 h dark) and food and water were provided *ad libitum*.

Neutropenia was achieved through 0.2 mL intraperitoneal (IP) administrations of cyclophosphamide 150 mg/kg on day minus 4 and an additional 100 mg/kg on day minus 1. A predictable degree of renal impairment was produced using 5 mg/kg of uranyl nitrate (Electron Microscopy Sciences, Hatfield, PA, USA) dissolved in sterile water administered as a 0.2 mL IP injection on day minus 3. Bacterial colonies from the overnight culture plate were suspended in normal saline to a McFarland target of 1.25 to produce the final inoculum. Mice were anaesthetised using inhaled isoflurane, manually restrained upright and infected with 50 µL of bacterial suspension via the nares. Each bacterial inoculation suspension was used within 30 minutes of initial preparation.

### Pharmacokinetic studies

#### Ex vivo tobramycin plasma protein binding studies

Escalating single doses of tobramycin (2.5, 5 and 10 mg/kg) were administered subcutaneously to determine tobramycin plasma protein binding. Triplicate pooled plasma and ultrafiltrate samples were collected at 1 h (5 mice per replicate, 15 mice per dose) and stored at −80°C until concentration determination. Whole blood was collected in K_2_EDTA tubes and then subsequently centrifuged at 4°C at 3000**g** for 10 minutes. Plasma was separated (total plasma) and 900 µL was added to an ultrafiltration device (Centrifree^®^, Merck Millipore Ltd, Ireland) and centrifuged using a fixed rotor at 10°C at 2000**g** for an additional 45 minutes to obtain protein free ultrafiltrate. The triplicate free and total tobramycin concentrations were averaged and then free fractions were calculated using the equation: free fraction = concentration_ultrafiltrate_/concentration_total plasma_.

#### Human-simulated exposure pharmacokinetic studies

The purpose of these studies was to establish HSRs in both plasma and ELF in the COMBINE murine neutropenic pneumonia model equivalent to clinical doses of meropenem (2 g every 8 h as 3 h infusion),^9^ cefiderocol (2 g every 8 h as 3 h infusion)^10^ and tobramycin (7 mg/kg as 0.5 h infusion)^11–13^ based on unbound (free) exposures. Human plasma protein binding values of 2%, 58% and 10%, respectively, were used to determine unbound human exposures.^14–16^ While the human plasma concentration–time profile for tobramycin is well described in the literature and made for clear exposure targets around which to develop a plasma HSR in the model, the available literature on the human ELF concentration–time profile was sparser and provided only point-to-point penetration estimates. Because overall AUC exposures between plasma and ELF are a better characterization of penetration, we fit a linear regression (*R*^2^ = 0.986) to single point penetration ratios at 0.5, 2, 4 and 8 h.^12^ Using the equation *C*_ELF_ = *C*_plasma_ ×  (0.1223 × time + 0.1567), a physiologically plausible human ELF exposure versus time profile was generated that yielded a AUC_ELF_/AUC_plasma_ penetration ratio of 0.69, which is similar to the 0.64 reported in a recent population PK analysis of tobramycin human ELF.^17^

**Figure 1.**
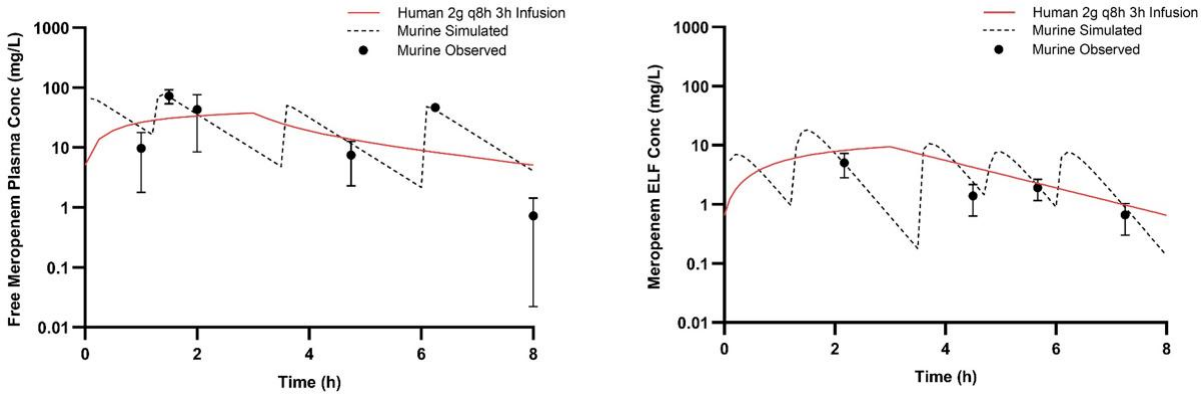
Humanized plasma and pulmonary ELF meropenem exposures after administration of respective HSRs in the COMBINE murine neutropenic pneumonia model. This figure appears in colour in the online version of *JAC* and in black and white in the print version of *JAC*.

**Figure 2.**
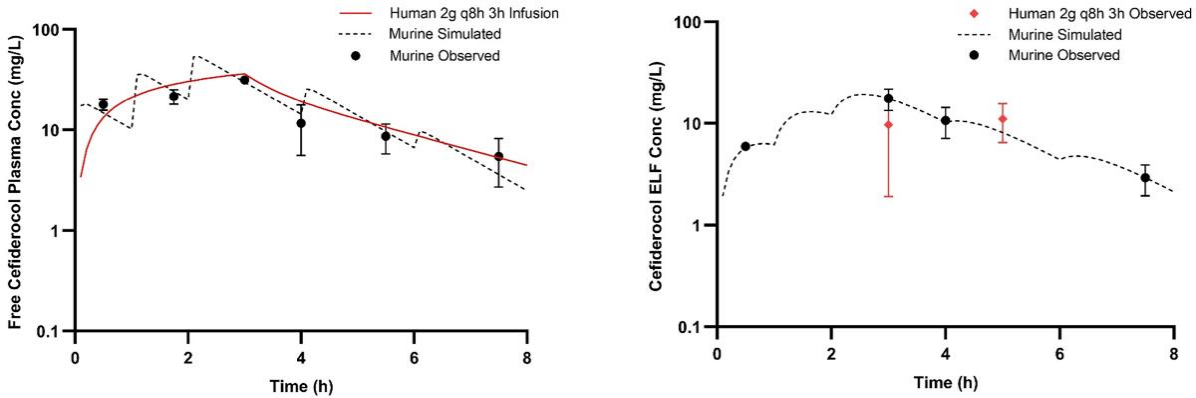
Humanized plasma and pulmonary ELF cefiderocol exposures after administration of respective HSRs in the COMBINE murine neutropenic pneumonia model. This figure appears in colour in the online version of *JAC* and in black and white in the print version of *JAC*.

**Figure 3.**
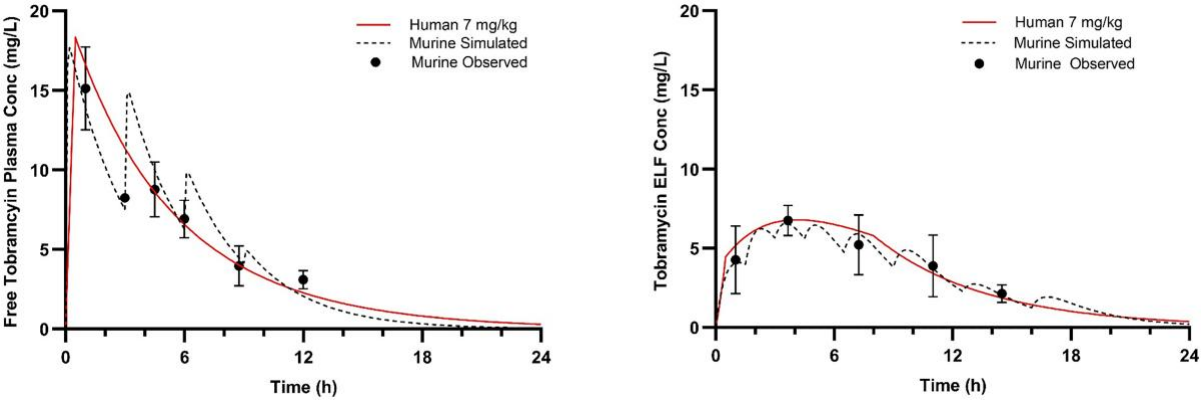
Humanized plasma and pulmonary ELF tobramycin exposures after administration of respective HSRs in the COMBINE murine neutropenic pneumonia model. This figure appears in colour in the online version of *JAC* and in black and white in the print version of *JAC*.

At predefined timepoints (shown in Figures 1–3), groups of six mice had blood sampled via retro-orbital bleeding (two time points, under anaesthesia with isoflurane 2%–3% v/v in 100% oxygen via inhalation) and/or cardiac puncture (one terminal time point). Proparacaine hydrochloride ophthalmic solution 0.5% was applied to the eye (1–2 drops) after blood sampling via retro-orbital bleeding. The volume of blood collected was 0.15 mL per sample via retro-orbital bleeding with subsequent fluid replacement using 0.2 mL normal saline given IP. Mice were euthanized by CO_2_ exposure before cardiac puncture. Following blood collection by cardiac puncture, but before cervical dislocation, bronchoalveolar lavage (BAL) fluid was collected from the mice at the same time points. A catheter was inserted into the trachea of the mice, and lungs were lavaged with four separate aliquots of 0.4 mL of normal saline. The BAL fluid was withdrawn immediately after injection and pooled for each animal. Pooled BAL was centrifuged for 10 min at 4°C and the supernatant was collected for analysis.

Total plasma concentrations were corrected to free using murine protein binding percentages determined presently for tobramycin, and previously determined values for meropenem (8%) and cefiderocol (31.6%).^18,19^ Drug concentrations in ELF were estimated by correcting the drug concentration in BAL fluid for the dilution with normal saline during lung lavage using the following formula: compound_ELF_ = compound_BAL_ × (urea_plasma_/urea_BAL_), where compound_BAL_ is the measured drug concentration in the BAL fluid sample and urea_plasma_ and urea_BAL_ are the concentrations of urea in paired plasma and BAL fluid samples from each mouse, respectively. Owing to the lack of albumin in BAL fluid, total ELF concentrations were considered unbound.

Statistical outliers for each respective analyte were removed by the interquartile range method. Previously reported meropenem, cefiderocol and plazomicin (an aminoglycoside similar to tobramycin) plasma HSRs developed in alternative murine models were used as a baseline.^19,20^ Mathematical modifications were made to the baseline regimens as necessary to achieve translational exposures in the COMBINE model and repeat confirmatory PK studies were undertaken for both plasma and ELF. A pharmacokinetic model was fit to the plasma and ELF concentrations of each compound and the best-fit estimate parameters were determined by nonlinear least-square techniques (WinNonlin, version 8.3, Pharsight Corp., Mountain View, CA, USA). Compartment model selection was based on visual inspection of the fit and the Akaike information criterion. The parameter estimates for each respective drug and matrix were used to calculate *f*AUC_0-24_, *f*C_max_ and %*f*T > MIC (meropenem and cefiderocol only) at each doubling dilution.

The sample size calculation was performed using nQuery Advisor based on the following: (i) the mean %CV of the PK parameter for typical antibiotics is usually <30%, and (ii) a two-sided 95% confidence interval with 80% probability will have an interval that extends no more than 1 SD from the observed mean. As a result, the sample size of six mice per time point is sufficient for the assessment of drug disposition.

### Analytical procedures

All compounds were analysed using a Waters Acquity UPLC H-Class system with tandem TQ-XS mass spectrometer (LC-MS/MS) equipped with an Acquity UPLC BEH C18 (1.7 μm, 2.1 × 50 mm) column maintained at 40°C and a sample manager cooled to 5°C unless otherwise stated. All reagents were obtained from commercial sources and used without further purification. Assay protocols were validated in accordance with Food and Drug Administration bioanalytical method validation guidelines.^21^ All samples above the limit of quantification were diluted with blank matrix before sample preparation. Mean interday coefficients of variance (CV) for each assay method are shown in Table 1.

**Table 1.** Mean interday CVs for each assay method

Analyte	Matrix	LQC (%)	MQC (%)	HQC (%)
Urea	Saline	9.9	9.6	11.5
Meropenem	Saline	12.4	3.5	4.2
	Plasma	9.2	3.5	5.8
Cefiderocol	Saline	2.7	3.5	3.5
	Plasma	10.8	4.1	1.6
Tobramycin	Saline	10.0	9.8	6.4
	Plasma	5.2	5.7	5.7

Urea concentrations in BAL and murine K_2_EDTA plasma were determined using standards in saline (5–500 μg/mL) and [^13^C]-urea as the internal standard. The internal standard was diluted with 3:4 acetonitrile:water to yield a 950 ng/mL solution of [^13^C]-urea for protein precipitation. Compounds were monitored using an ESI probe in positive acquisition mode. The quantitative mass transition for urea was 61.0 → 43.8. The quantitative mass transition for [^13^C]-urea was 62.0 → 45.0. For the preparation of all standards and samples, 630 μL of protein precipitation solution was added to a well plate containing 30 μL of standard or sample. Nominal concentrations of the low quality control (LQC), medium quality control (MQC) and high-quality control (HQC) for urea in saline were 7.5, 75 and 400 μg/mL, respectively.

Concentrations of meropenem in murine K_2_EDTA plasma (0.01–100 μg/mL) and saline (0.01–100 μg/mL) were determined using meropenem-d_6_ as the internal standard. The internal standard was diluted with 9:1 acetonitrile:water to yield a 50 ng/mL solution of meropenem-d_6_ for protein precipitation. Compounds were monitored using an ESI probe in positive acquisition mode. The quantitative and qualitative mass transitions for meropenem were 384.2 → 141.2 and 384.2 → 320.17, respectively. The quantitative mass transition for meropenem-d_6_ was 390.2 → 147.0. For the preparation of plasma standards and samples, 250 μL of protein precipitation solution was added to a microcentrifuge tube containing 50 μL of standard or sample. The suspension was vortexed to mix and centrifuged at 15 000**g** for 2 minutes. For analysis, 50 μL of supernatant transferred to a 96-well plate and diluted with 200 μL of water. For the preparation of saline standards and BAL samples, 125 μL of protein precipitation solution was added to a well plate containing 25 μL of standard or sample. This mixture was then diluted with 600 μL of water. Nominal concentrations of the LQC, MQC and HQC for meropenem in both matrices were 0.025, 1 and 50 μg/mL, respectively.

Concentrations of cefiderocol in murine K_2_EDTA plasma (0.1–100 μg/mL) and saline (0.025–100 μg/mL) were determined using cefiderocol-d_8_ as the internal standard. The internal standard was diluted with 9:1 acetonitrile:water to yield a 100 ng/mL solution of cefiderocol-d_6_ for protein precipitation. Compounds were monitored using an ESI probe in positive acquisition mode. The quantitative and qualitative mass transitions for cefiderocol were 752.2 → 285.0 and 752.2 → 214.1, respectively. The quantitative mass transition for cefiderocol-d_8_ was 760.3 → 293.0. Cefiderocol standards and samples were prepared in the same manner as described for meropenem in plasma and saline previously. Nominal concentrations of the LQC, MQC and HQC for cefiderocol in both matrices were 0.15, 1 and 50 μg/mL, respectively.

Concentrations of tobramycin in murine K_2_EDTA plasma (0.5–100 μg/mL) and saline (25–10 000 ng/mL) were determined using tobramycin-d_5_ as the internal standard and a Kinetex F5 (2.6 um, 150 × 2.1 mm) column maintained at 40°C. Compounds were monitored using an ESI probe in positive acquisition mode. The quantitative and qualitative mass transitions for tobramycin were 468.2 → 163.4 and 468.2 → 324.0, respectively. The quantitative mass transition for tobramycin-d_5_ was 479.5 → 331.0. For the preparation of plasma standards and samples, the internal standard was diluted with 9:1 acetonitrile:water to yield a 500 ng/mL solution of tobramycin-d5 for protein precipitation. To a microcentrifuge tube containing 50 μL of standard or sample, was added 250 μL of protein precipitation solution. The suspension was vortexed to mix and centrifuged at 15 000**g** for 2 minutes. For analysis, 50 μL of supernatant transferred to a 96-well plate and diluted with 200 μL of water containing 3% (v/v) formic acid in water. Nominal concentrations of the LQC, MQC and HQC for tobramycin in plasma were 0.75, 7.5 and 75 μg/mL, respectively. For the preparation of saline standards and BAL samples, the internal standard was diluted with water to yield a 500 ng/mL solution of tobramycin-d_5_. To a well plate containing 25 μL of standard or sample was added 25 μL of tobramycin-d_5_ solution and 200 μL of water containing 2.5% (v/v) trichloroacetic acid. Nominal concentrations of the LQC, MQC and HQC for tobramycin in saline were 75, 500 and 7500 ng/mL, respectively.

## Results

### Pharmacokinetic studies

#### Ex vivo tobramycin serum protein binding studies

Mean percentages of protein binding (±standard deviation) were 27.1% (2.9%), 20.5% (9.3%) and 19.8% (1.5%) at the 1-h timepoint after receiving doses of 2.5, 5 and 10 mg/kg, respectively. These plasma protein binding percentages were considered exposure-independent across the studied dose range, and thus the average observed value across the three doses of 22.4% (77.6% unbound) was applied uniformly to correct for free tobramycin plasma concentrations. Protein binding in humans, while often considered clinically negligible, has been shown to be ∼10%.^16^

#### Human-simulated exposure pharmacokinetic studies

The observed meropenem, cefiderocol and tobramycin plasma and ELF concentrations were satisfactorily described using one-compartment pharmacokinetic models with first order elimination. The best-fit parameters for each drug in each matrix are displayed in Tables 2 and 3. Importantly, the pharmacokinetic parameters coupled with interspecies differences in protein binding did not allow for utilization of the same murine dosing regimen to appropriately simulate humanized exposures in both matrices for any of the three drugs investigated. Therefore, separate dosing regimens were required for each individual matrix of each compound. The murine dosing regimens administered to achieve humanized exposures of each of the three compounds in both matrices are listed in Table 4. Owing to the increased lung penetration observed in the murine model for both meropenem and cefiderocol compared to humans, humanizing the ELF required a 35%–40% reduction in the total drug delivered relative to the plasma HSR. By contrast, due to lower ELF penetration of tobramycin in mice, a 35% increase in total drug delivered was required to approximate the ELF exposure in man. The murine plasma and ELF concentration–time profiles after administration of respective plasma and ELF HSRs for each compound are presented in Figures 1–3. Using the interquartile range method, 10/108 (9.3%) plasma samples and 7/78 (9.0%) BAL samples were excluded from final pharmacokinetic analyses.

**Table 2.** Best-fit plasma pharmacokinetic parameters in the COMBINE murine neutropenic pneumonia model

Drug	*V*_plasma_ (L/kg)	*K*_a_ (1/h)	*K*_el_ (1/h)
Meropenem	0.04	1.34	27.13
Cefiderocol	0.19	21.71	0.74
Tobramycin	0.37	23.68	0.31

*V*_plasma_, volume of distribution in central compartment; *K*_a_, absorption rate constant into central compartment; *K*_el_, elimination rate constant from central compartment.

**Table 3.** Best-fit ELF pharmacokinetic parameters in the COMBINE murine neutropenic pneumonia model

Drug	*V*_ELF_ (L/kg)	*K*_a-ELF_ (1/h)	*K*_el-ELF_ (1/h)
Meropenem	1.60	7.00	2.50
Cefiderocol	0.33	1.94	0.72
Tobramycin	0.79	1.74	0.35

*V*_ELF_, volume of distribution in ELF compartment; *K*_a-ELF_, absorption rate constant into ELF compartment; *K*_el-ELF_, elimination rate constant from ELF compartment.

**Table 4.** Dosing regimens administered to achieve humanized exposures in plasma and ELF

Human regimen	Matrix	Murine regimen (dorsal subcutaneous injections in 0.1 mL)
Meropenem 2 g every 8 h as a 3 h infusion	Plasma	65 mg/kg at 0 h, 65 mg/kg at 1.25 h, 45 mg/kg at 3.5 h, 45 mg/kg at 6 h repeated every 8 h
	ELF	20 mg/kg at 0 h, 50 mg/kg at 1.25 h, 30 mg/kg at 3.5 h, 20 mg/kg at 4.75 h, 15 mg/kg at 6 h repeated every 8 h
Cefiderocol 2 g every 8 h as a 3 h infusion	Plasma	5 mg/kg at 0 h, 7.5 mg/kg at 1 h, 10 mg/kg at 2 h, 3.5 mg/kg at 4 h, 1 mg/kg at 6 h repeated every 8 h
	ELF	3.75 mg/kg at 0 h, 5 mg/kg at 1 h, 6.25 mg/kg at 2 h, 1.75 mg/kg at 4 h, 1 mg/kg at 6 h repeated every 8 h
Tobramycin 7 mg/kg as a 30 min infusion	Plasma	9 mg/kg at 0 h, 4 mg/kg at 3 h, 2 mg/kg at 6 h, 0.5 mg/kg at 9 h
	ELF	4.8 mg/kg at 0 h, 3.6 mg/kg at 1.5 h, 2.4 mg/kg at 3 h, 2.4 mg/kg at 4.5 h, 2.7 mg/kg at 6.5 h, 2.4 mg/kg at 9 h, 1.2 mg/kg at 12.5 h, 1.2 mg/kg at 16 h

The %*f*T > MIC at relevant doubling dilutions achieved in the model with separate HSRs to characterize the plasma and ELF of meropenem and cefiderocol are appropriately matched to average exposures in humans receiving 2 g every 8 h as 3 h infusions as shown in Table 5. For tobramycin, the *f*C_max_ and *f*AUC_0-24_ achieved with both the plasma and ELF HSRs were well matched to humans receiving 7 mg/kg. The human plasma *f*C_max_ and *f*AUC_0-24_ compared with murine plasma were 18.3 versus 17.7 mg/L and 103 versus 103 mg h/L, while the human to murine ELF were 6.8 versus 6.6 mg/L and 79 versus 76 mg h/L, respectively.

**Table 5.** Comparison of %*f*T > MIC values achieved with meropenem and cefiderocol in both plasma and ELF at each MIC in humans and in mice receiving respective humanized regimens

			%*f*T > MIC for a MIC (µg/mL) of:							
Drug	Matrix	Species	1 (%)	2 (%)	4 (%)	8 (%)	16 (%)	32 (%)	64 (%)	128 (%)
Meropenem	Plasma	Mouse^a^	100	100	94	75	55	29	5	0
		Human^b^	100	100	100	78	50	19	0	0
	ELF	Mouse^c^	78	62	43	14	3	0	0	0
		Human^b^	90	71	50	16	0	0	0	0
Cefiderocol	Plasma	Mouse^a^	100	100	91	76	50	16	0	0
		Human^b^	100	99	98	76	48	11	0	0
	ELF	Mouse^c^	100	100	85	50	14	0	0	0
		Human^b^	Minimally characterized, see Figure 2							

^a^Average exposures achieved using the plasma HSRs listed in Table 4.

^b^Average exposures achieved with 2 g every 8 h as a 3 h infusion.

^c^Average exposures achieved using the ELF HSRs listed in Table 4.

## Discussion

Characterization of lung ELF is critical for the clinical translation of preclinical animal pneumonia models. Utilizing the standardized COMBINE protocol for the murine neutropenic pneumonia model, we developed and confirmed HSRs in both plasma and ELF for meropenem, cefiderocol and tobramycin: three antibiotics commonly used to treat critically ill patients with pneumonia. Different dosing regimens were required for each matrix, resulting in a total of six unique HSRs across the three compounds, with the general understanding that HSRs based on ELF exposures provide enhanced translation to the clinic relative to plasma HSRs when interspecies differences in target site penetration exist. It should be noted that all these regimens were developed in the model with mice infected with a singular strain. Alternative isolates may have differing levels of virulence and propensity to cause septic shock, which could result in some alteration in the renal clearance of these compounds. However, as has been demonstrated previously, these changes can generally be considered negligible and fall well within the expected variability seen in humans.^22^

While human plasma concentration–time profiles are often well characterized, ELF profiles, especially in infected patients, are often inadequately defined. Existing literature for meropenem ELF pharmacokinetics in patients with ventilator-associated pneumonia provided sufficient %*f*T > MIC exposure targets to allow for the development of an ELF HSR.^9^ On the other hand, cefiderocol ELF exposures in mechanically ventilated patients with pneumonia using the clinical dose of 2 g every 8 h as a 3 h infusion have been sparsely characterized with two sampling timepoints at 3 h (end of infusion) and 5 h.^10^ Based on these limited data, it could be reasonably concluded that cefiderocol ELF exposures are likely to remain above the susceptibility breakpoint of 4 mg/L for a sufficient percentage of the dosing interval to predict efficacy, which was recapitulated with the cefiderocol ELF HSR. Similarly, because aminoglycoside activity is described by *C*_max_/MIC and *f*AUC/MIC, we needed to estimate these values in tobramycin human ELF through applying a regression based on single point-to-point penetration estimates from plasma over time to predict an ELF concentration–time curve. While this extrapolation was performed with one external pharmacokinetic dataset, the AUC_ELF_/AUC_plasma_ penetration ratio of 0.69 was consistent with the result of 0.64 reported in a recent population PK study.^17^ It is imperative that future PK studies undertaken for novel compounds seeking an indication for pneumonia characterize lung exposures throughout the entire dosing interval. Otherwise, preclinical models seeking to simulate humanized exposures will continually rely on methods that are imprecise and based on many assumptions.

Our development of humanized exposure profiles for both plasma and ELF in the well-defined COMBINE murine neutropenic pneumonia model provides a translational link between preclinical studies, *in vitro* potency and the clinical efficacy of meropenem, cefiderocol and tobramycin for the treatment of Gram-negative pneumonia. Furthermore, the application of these defined HSRs in the COMBINE model will allow for the establishment of quantitative bacterial density benchmarks to better delineate the potential clinical utility of novel therapies relative to accepted standard of care treatments.^22^
